# Refeeding syndrome

**DOI:** 10.4103/0972-5229.43683

**Published:** 2008

**Authors:** Swagata Tripathy, Padmini Mishra, S. C. Dash

**Affiliations:** **From:** Department of Anaesthesia and Critical Care, Kalinga Institute of Medical Sciences Medical College, Bhubaneswar, India

**Keywords:** Hypophosphatemia, malnutrition

## Abstract

We report a case of a fifty-year-old male who was admitted with a three month history of increasing weakness, prostration, decreasing appetite and inability to swallow. The patient was a chronic alcoholic, unemployed, and of very poor socioeconomic background. The patient was initially investigated for upper GI malignancy, Addisons disease, bulbar palsy and other endocrinopathies. Concurrent management was started for severe electrolyte abnormalities and enteral nutritional supplementation was begun. By the fourth day of feeding patient developed severe hypophosphatemia and other life-threatening features suggesting refeeding syndrome. The patient was managed for the manifestations of refeeding syndrome. A final diagnosis of chronic alcoholic malnutrition with refeeding syndrome was made. Refeeding of previously starving patients may lead to a variety of complications including sudden death.

## Introduction

Severely malnourished patients can undergo life-threatening fluid and electrolyte shifts following the initiation of aggressive nutritional support therapy. Refeeding syndrome[1] is a condition known to occur in previously fasting / malnourished patients who have been started suddenly on high calorie carbohydrate rich diet. It may manifest with myriad manifestations like cardio respiratory failure, neuromuscular manifestations, seizures and sudden death. Anecdotal case reports in literature suggest the serious, sometimes fatal nature of this condition if not anticipated or managed promptly.[2]

The purpose of this report is to highlight the need of anticipating the occurrence of this under-recognised but treatable condition. We review the other causes of refeeding syndrome and the management in Indian/other scenario where intravenous or readymade oral phosphate preparations are not available.

## Case Report

A fifty-year-old man presented with a history of gradually increasing anorexia, weakness, and prostration of three months duration. He complained of progressive difficulty in swallowing which was more for solids. Paraesthesia in hands and legs were also present. He had a tribal origin and was of extremely poor socioeconomic status. He was an uneducated and unemployed, chronic alcoholic.

On examination, the patient was cachectic (BMI 15 kg/m^2^), confused, and severely dehydrated. Vitals recorded showed a pulse rate of 50 bpm (ECG showed a first degree heart block) and right arm supine blood pressure of 70/50 mmHg. He was resuscitated with intravenous crystalloids and the hemodynamic status improved.

Routine hematology and biochemistry investigations were done. A severe electrolyte imbalance with elevated serum urea and creatinine levels was found [Table 1]. Other investigations, Chest X-Ray and electrocardiogram were essentially within normal limits.

**Table 1:** Biochemical parameters

Parameters	Normal	Day	1	2	3	4	5	6	7	10	15
Urea (mg/dl)	20-40		50	48	47	51	42	40	32	27	18
Creatinine (mg/dl)	0.9-1.2		2.1	1.8	1.8	2.0	1.9	1.6	1.4	1.1	1.2
K (mmol/l)	3.5-4.5		1.18	3.3	3.6	3.7	2.6	3.9	3.7	4.5	3.8
Calcium (meq/dl)	9.0-11.0		6.1	7.0	8.5	5.0	6.5	6.5	8.0	9.0	8.8
Phosphate (meq/dl)	3.0-5.0				4.9		4.3	1.6	1.9	2.1	3.1
Magnesium (mmol/dl)	1.5-2.5				1.6						2.4
Na (mmol/l)	135-145		131	149	157	154	150	147	147	148	148
Albumin (gm/dl)	3.5-5.0				2.8						3.2
INR	1.0-1.5								3.2		1.2

Patient was managed symptomatically with fluid, electrolyte and vitamin supplementation. With the patients history in mind he was investigated for upper GI malignancy, Addisons disease (suspected tubercular etiology common in our part of the country), bulbar palsy, and thyroid dysfunction (hypoparathyroidism). Upper GI endoscopy, ultrasound abdomen, serum cortisol assay, PCR for *Mycobacterium tuberculosis*, contrast enhanced CT scan of head, serum parathormone and TSH levels were also done. All investigations were within normal parameters. A working diagnosis of chronic alcoholic malnutrition with severe dyselectrolytemia was made.

Patient showed some improvement in muscle power with the correction of electrolytes in the first two days during which he was on intravenous fluids. Third day onwards low calorie(10-15 kcal/kg) carbohydrate restricted feeds through a Ryle's tube were started. The low volume feeds given fourth hourly were well accepted.

On the fourth day (sixth day of hospital stay) after initiation of therapy (refeeding) he developed profound weakness (flaccid paralysis), paraesthesias and carpopedal spasms of all four limbs. Psychiatric manifestations like hallucinations were observed. Onset of coagulopathy (INR 3.2) and diarrhoea was noted. A provisional clinical diagnosis of refeeding syndrome was arrived at, based on the history, examination and biochemical results [Table 1].

Prompt management was initiated with intravenous infusions of potassium, calcium, magnesium and thiamine. Proctoclysis – (phosphate containing enema) delivered through a Ryle's tube was resorted to as the intravenous form is unavailable in our country. Massive potassium replacements amounting to 40 meq/hour were given through a central intravenous line. Calcium was initially given intravenously, and subsequently the patient was stabilized on 3 gm of Calcium in divided doses per day orally. Phosphate was supplemented with a maximum of two Process Proctoclysis enema bags (100ml of w/v of 10%sodium hihydrogen phosphate and 8%disodium hydrogen phosphate) enterally for three days till serum phosphate levels reached 3 meq/l. Frequent monitoring of the serum levels of the above electrolytes was adhered to and managed as required.

A remarkable clinical improvement in the paraesthesias and carpopedal spasms was observed by the eighth day. The renal parameters normalized over five days. He was also able to swallow food from the eighth day. The patient was finally discharged fully ambulatory on the fifteenth day with oral potassium, calcium and vitamin supplements advised along with a balanced diet.

## Discussion

Refeeding[1] syndrome is well-recognized in patients with starvation and anorexia nervosa, those with chronic alcoholism and post operative patients who receive total parenteral nutrition.[3] It is also seen early in the treatment of diabetic hyperosmolar states.[4] It has less frequently been recognized in malnourished in patients receiving enteral nutritional support.[5]

The pathophysiology of refeeding syndrome has now been elucidated. In starvation with reduced intake of carbohydrates the secretion of insulin is decreased. Lipolysis and body protein breakdown occurs to produce energy. This results in a loss of intracellular stores of electrolytes specially phosphate. Serum levels may appear normal in spite of severe total body store depletion due to shift in intra cellular phosphate. In these conditions sudden aggressive refeeding with carbohydrates leads to an insulin surge with consequent intracellular shift in electrolytes especially potassium and phosphate.

Serum phosphate levels of less than 1.5 meq/l (normal levels 3-5 meq/l) can produce the clinical features of refeeding syndrome which include rhabdomyolysis, leukocyte dysfunction, respiratory failure, cardiac failure, hypotension, arrhythmias, seizures, coma and sudden death. The early features of hypophosphatemia are non specific and may go unrecognized.[6] Therefore awareness of this syndrome and a high index of suspicion are mandated for early and effective management of this potentially life threatening condition.

This phenomenon typically manifests on the fourth day after commencement of feeding. The anabolic milieu results in manifestation of deficiencies of thiamine (encephalopathy, confusion, heart failure), phosphate (deficiency of phosphorylation byproducts like ATP leading to muscle weakness, hematological effects), potassium (arrhythmias, sudden death), sodium and water retention (congestive cardiac failure).

In addition to these consequences of hypophosphatemia, low levels of potassium and magnesium predispose to arrhythmias, constipation and paralytic ileus, fasciculations, paraesthesias, confusion, neurologic manifestations, and impaired renal concentrating abilities.[7]

Our patient displayed the hallmark dip in serum phosphate levels on the fourth day of refeeding. He developed neuromuscular complications, coagulopathy (prolonged PTT) and psychiatric complaints, sodium retention and loose stools which resolved with dietary and other replacement maneuvers as mentioned. Anticipation of this complication and early diagnosis thereof helped prompt intervention halted the downhill course in our patient with a fast recovery to normalcy.

No randomized controlled trials of refeeding syndrome have been performed and the optimal regime therefore needs to be determined. Treatment of refeeding syndrome can be assisted by the hospital nutrition team. Patients at-risk for malnutrition or developing refeeding syndrome need to be identified. Enteral or parenteral feeding should be started at a reduced calorific rate (25-50% of daily caloric needs). Carbohydrate intake should be limited. Serum phosphate, magnesium, calcium, potassium, sodium, urea and creatinine levels should be measured and supplementation should be done as needed. Feeding may be stopped and restarted after 24h at a lower rate if symptoms of cardiac failure like tachycardia and tachypnoea develop.

Intravenous phosphate preparations are not available in our country. Therefore an enteral route of phosphate supplementation can be used as seen with our experience. Proctoclysis enema is an easily available and rich source of phosphate when administered via the Ryles tube. We administered a maximum of three standard enema pouches per day for three days along with laboratory monitoring. The rise in phosphate levels was satisfactory.

Awareness about refeeding syndrome, its at-risk population, early diagnosis and prompt supportive and specific intervention is of paramount importance in managing this rare condition.

### Key points

- Refeeding syndrome is characterized primarily by neurological, muscular and hematological changes.
- These changes are associated with profound falls in serum levels of phosphate, potassium magnesium, thiamine etc.
- Refeeding syndrome can occur when nutrition in the form of carbohydrate (enteral or parenteral) is reintroduced following starvation.
- Tachypnea and tachycardia may be useful early clinical signs of refeeding syndrome developing in a patient who has been renourished after a period of starvation.
- Typical laboratory finding is development of hypophosphatemia on the fourth day of refeeding.
